# Efficient virtual high-content screening using a distance-aware transformer model

**DOI:** 10.1186/s13321-023-00686-z

**Published:** 2023-02-08

**Authors:** Manuel S. Sellner, Amr H. Mahmoud, Markus A. Lill

**Affiliations:** Department of Pharmaceutical Sciences, University of Basel, Basel, Switzerland

**Keywords:** Virtual screening, Similarity search, Deep learning, Transformer model

## Abstract

Molecular similarity search is an often-used method in drug discovery, especially in virtual screening studies. While simple one- or two-dimensional similarity metrics can be applied to search databases containing billions of molecules in a reasonable amount of time, this is not the case for complex three-dimensional methods. In this work, we trained a transformer model to autoencode tokenized SMILES strings using a custom loss function developed to conserve similarities in latent space. This allows the direct sampling of molecules in the generated latent space based on their Euclidian distance. Reducing the similarity between molecules to their Euclidian distance in latent space allows the model to perform independent of the similarity metric it was trained on. While we test the method here using 2D similarity as proof-of-concept study, the algorithm will enable also high-content screening with time-consuming 3D similarity metrics. We show that the presence of a specific loss function for similarity conservation greatly improved the model’s ability to predict highly similar molecules. When applying the model to a database containing 1.5 billion molecules, our model managed to reduce the relevant search space by 5 orders of magnitude. We also show that our model was able to generalize adequately when trained on a relatively small dataset of representative structures. The herein presented method thereby provides new means of substantially reducing the relevant search space in virtual screening approaches, thus highly increasing their throughput. Additionally, the distance awareness of the model causes the efficiency of this method to be independent of the underlying similarity metric.

**Supplementary Information:**

The online version contains supplementary material available at 10.1186/s13321-023-00686-z.

## Introduction

### Molecular similarity search

The mean financial burden of researching and developing a new drug has been estimated to exceed 1 billion US dollars [1]. Resource, cost, and time efficient methods of finding new drug molecules are therefore imperative for reducing the cost and duration of drug development. Using computer-based methods can help reach this goal.

A well-known concept in drug development is that similar molecules exhibit similar properties and activity profiles [2, 3]. This can enable researchers to find novel hits by comparing them with known active substances, which is the main principle behind similarity search in drug development. Similarities between compounds can be determined by different strategies, from simple descriptor-based comparisons over 2D fingerprints to detailed 3D measures such as shape-based or field-based similarities dependent on alignment of the molecules to be compared. To calculate similarities between molecules for large-scale similarity search, typically molecular fingerprints are utilized and computed. These fingerprints encode chemical properties and usually consist of binary vectors. While traditional molecular fingerprints were mainly rule-based (e.g. based on the presence of substructures or atom-pairs [4, 5]), data driven fingerprints (e.g. learned by machine learning models) became more prominent in recent years [6]. Various metrics like the Tanimoto or Dice coefficient, or the Tversky index can be used to compute similarities based on these binary fingerprints [3].

There is a large variety of molecular fingerprints, ranging from simple fragment-based 2D methods to complex 3D approaches [2, 7]. 2D based fingerprints can easily be applied to virtual screenings of multi-million compound databases (up to several billion) [8, 9]. While this is possible in a relatively short period of time due to their low complexity, more complicated 3D similarity measures such as shape screening and similarity based on field points are realistically only feasible to use on smaller datasets of several hundred thousands up to a few million compounds [10, 11].

Here, we present a different approach to the problem of high-content similarity screening combining transformer-based autoencoder models, similarity-based latent space shaping, and direct sampling in the reduced latent space representation. In this current proof-of-concept study presented here, we demonstrate the feasibility of the approach using 2D fingerprint similarities. We show that our approach can capture molecular similarities very well in latent space. The performance of the presented model is, however, independent of the used similarity metric. This allows researchers to train a model on highly complex 3D similarity metrics and thus perform high-content screening using metrics that otherwise would not be feasible to apply to a large set of compounds. Since the presented problem falls under the domain of distance metric learning [12, 13], we show how to overcome this obstacle by implementing a custom loss function specifically designed to map similarities to Euclidian distances.

### Related work

Since the goal of this project is to group similar samples closer together in latent space while pushing dissimilar samples further apart, it shares similarities with contrastive learning approaches [14, 15]. Contrastive learning has been widely used in visual learning with great success [16–18]. Recently, it has also been applied to molecular data, not only in a supervised but also in a self- or unsupervised fashion [19–21]. Self-supervised methods have the advantage that they do not rely on the explicit labeling of positive (similar) and negative (dissimilar) samples. When it comes to molecular data, self-supervision is feasible in 2D space by slightly altering substructures of molecules to obtain positive samples. However, when moving to 3D representations, altering substructures may lead to large differences in the 3D conformation of a molecule, where it is not guaranteed that the newly generated structure is still similar to the original. Furthermore, our approach differs from contrastive learning by providing a continuous measure of similarities to allow for a ranking of molecules according to their similarity to a template.

The use of deep learning models to create latent space embedding of molecules is not novel and has been used for several years now [22, 23]. However, to our knowledge, this is the first time that the generated latent space was explicitly shaped in a way that allows the direct conservation of molecular similarities without having to rely on the discrimination of the data into different classes and without losing the direct scalability to higher dimensional representations.

A well established approach of learning chemical properties of molecules is by using so called autoencoders [24–27]. An autoencoder is a model that attempts to encode its input into latent space and decodes it again while minimizing the difference between the input and the decoded output. The latent space can be considered a reduced representation of the underlying structures of the chemicals in the dataset. Herein, we make use of an autoencoder in order to learn similarities of molecules. Honda et al. previously used a transformer model to generate molecular fingerprints from SMILES strings using a simple reconstruction loss function [24]. Bjerrum et al. found that mapping enumerated to canonical SMILES improves the conservation of similarities in latent space [25].

As mentioned before, conserving similarities in latent space is not only of high relevance in drug discovery but also in other fields such as image recognition. Schroff et al. [28] proposed a loss function called triplet loss (Eq. 1) which can be used to map related images to similar regions in latent space while increasing the distance between dissimilar images:

1 $$\begin{aligned} L(A,P,N) = max(||f(A) - f(P)|| - || f(A) - f(N) || + m, 0) \end{aligned}$$

This loss function relies on the definition of an anchor (*A*), a positive (i.e. similar) sample (*P*), and a negative (i.e. dissimilar) sample (*N*) and is therefore well suited for data with discrete labels. $f(\cdot )$ describes the coordinates of a compound in latent space, $||\cdot ||$ the L2-norm, and *m* the hyperparameter specifying a margin to separate similar from non-similar molecules.

In this work, we follow the approach of Honda et al. and use a transformer model to autoencode SMILES strings to generate fingerprints suitable for similarity calculations [24]. We then use the generated latent space encodings for similarity search based on Euclidian distances. In order to improve the similarity conservation in latent space, we compare a model based only on a reconstruction loss with models trained on additional loss terms to specifically learn similarities. Since the triplet loss function in Eq. 1 requires discrete labels, working with similarities requires the definition of a similarity threshold separating similar molecules from dissimilar ones. As such a separation is highly ambiguous for diverse sets of molecules, we developed a novel loss function which we call the similarity loss function. The similarity loss function can be used to work with continuous data, rendering it well-suited for working with similarities.

The herein presented models are therefore intended to estimate similarities based on Euclidian distances in latent space, allowing the subsequent use of exhaustive similarity search on a drastically reduced search space. We also show that a model trained on a small dataset is able to generalize to huge compound libraries containing highly diverse structures.

## Methods

### Model architecture

**Fig. 1:**
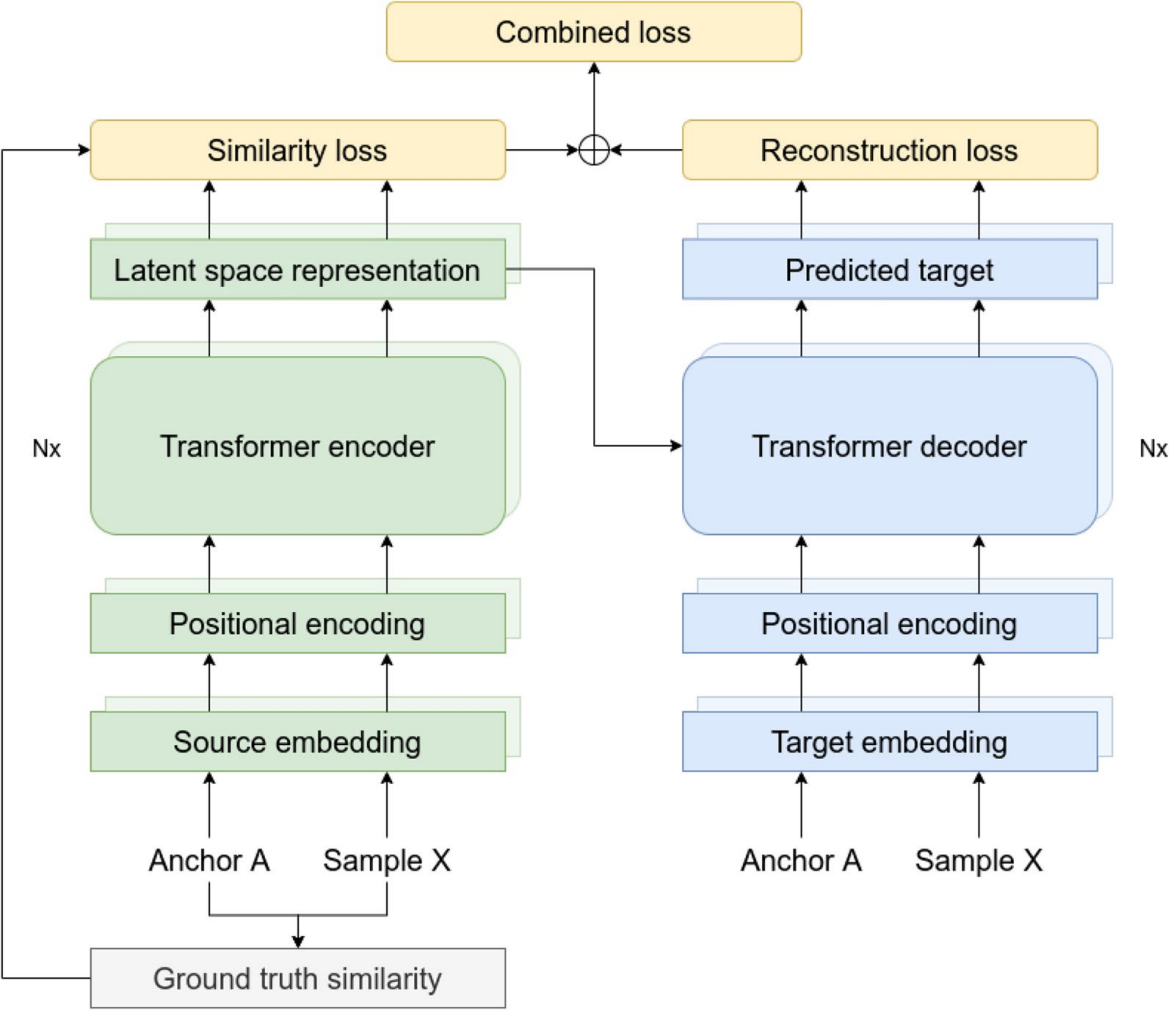
Architecture of the used transformer model. Encoder and decoder layers are constructed following the original publication of the transformer model by Vaswani et al. [34]. To help conserve similarities in latent space, a special loss function denoted as ”similarity loss” is added to the reconstruction loss

In recent years, transformer-based models witnessed great success in various areas such as natural language processing, speech recognition, object detection, and more [29–33]. In this work, we follow the initial transformer model architecture proposed by Vaswani et al. [34]. Figure 1 shows a representation of the implemented model architecture.

To encode simple SMILES representations of molecules, we first tokenized the strings, embedded them and added a positional encoding. An example of a tokenized SMILES string can be found in Additional file 1: Fig. S1−S3. The positional encoding is done using a set of sine and cosine functions of varying frequencies as indicated in Eq. 2 where *pos* refers to the position of the token in the sequence, *d* is the size of the embedding, and *i* is the dimension of the embedding. In this study, we set $d=256$.

2 $$\begin{aligned} \begin{aligned} PE(pos, 2i) = sin\bigg (\frac{pos}{10000^\frac{2i}{d}}\bigg ) \\ PE(pos, 2i+1) = cos\bigg (\frac{pos}{10000^\frac{2i}{d}}\bigg ) \end{aligned} \end{aligned}$$

The pre-processed data are then passed to a transformer encoder consisting of four layers. Each layer contains a multi-head attention layer. In this model, we used four heads per attention layer. To compute the attention, we follow the original article where attention is defined as shown in Eq. 3 where *Q*, *K*, and *V* are matrices containing the queries, keys, and values, respectively, and $d_k$ is the dimensionality of the keys [34].

3 $$\begin{aligned} attention(Q, K, V) = softmax\bigg (\frac{QK^T}{\sqrt{d_k}}\bigg )V \end{aligned}$$

This encoder computes a latent space representation of the input. To obtain a single vector representation for each source molecule, we average over all tokens in the sequence. For the decoder part, we feed the tokenized target SMILES to an embedding layer and add a positional encoding the same way it was done for the encoder part. Note that since we are working with an autoencoder, the source and target represent the same SMILES string while the target is right shifted. This means that the matrices containing the queries, keys, and values (Eq. 3) all contain the same information consisting of the tokenized SMILES strings. The queries and keys are used to calculate attention weights which represent the importance of each element in the SMILES string. These attention weights can then be used to compute a weighted sum of the values. The transformer decoder layers combine the predicted latent space representation of the source with the attention weights and masked target embeddings, and subsequently predict the target sequence.

**Fig. 2:**
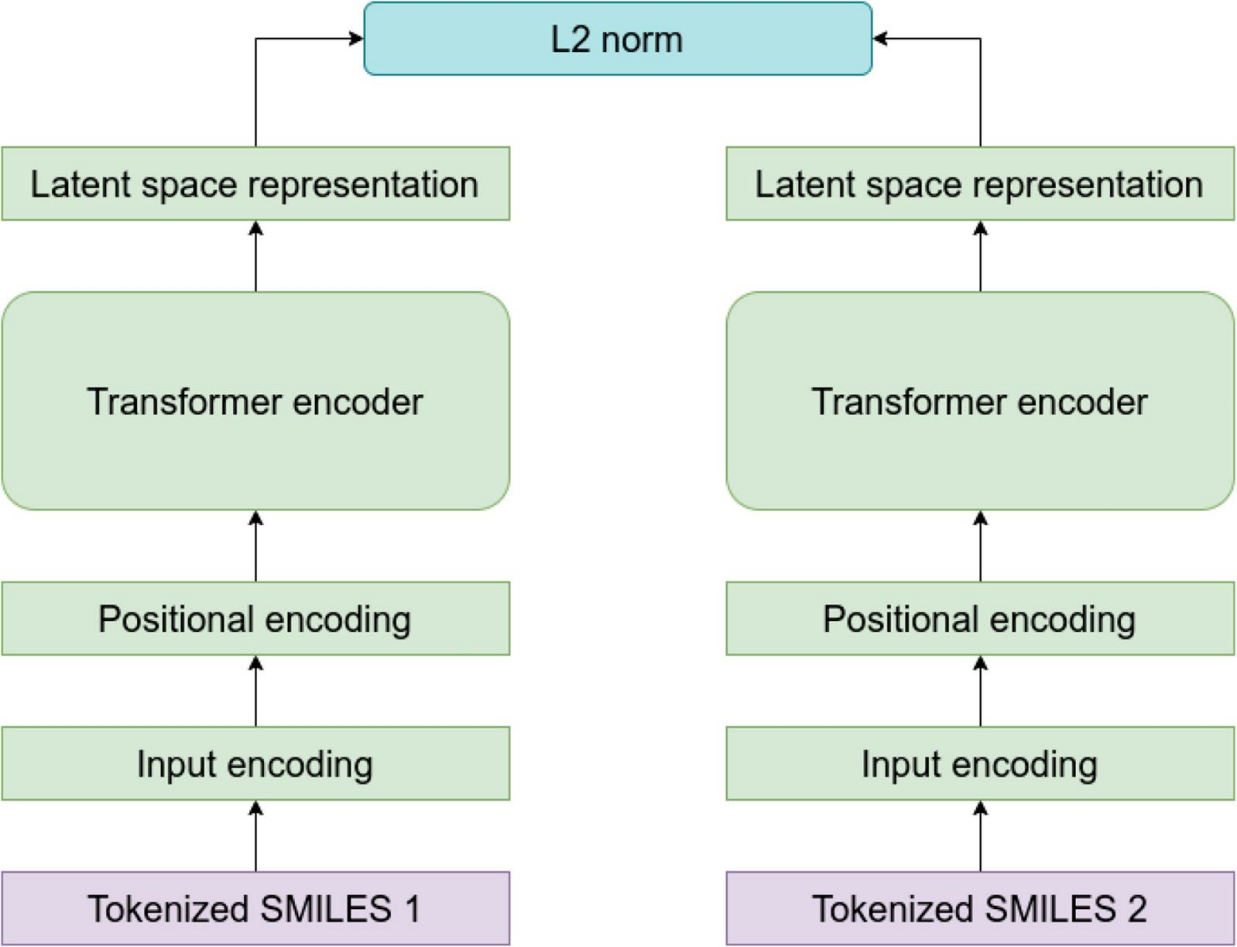
Predicting similarities between two molecules. The L2 norm is used to calculate the distance in latent space based on tokenized SMILES strings

In a regular transformer model, this prediction is then used to calculate the reconstruction loss usually in form of a cross entropy loss which is used to train the model. Here, we develop and test novel loss functions to conserve similarities in the produced latent space. When applying the model to predict similarities, the decoder part of the model will not be used. Similarities are calculated based solely on the latent space representation of the query molecules; the L2 norm is used to calculate the distance between two molecules in latent space (Fig. 2). In praxis, a perfect correlation between latent space distance and ground truth similarity metric cannot be expected. Therefore, the purpose of this model is to obtain high enrichment in predicted, similar compounds to reduce the relevant search space by a significant degree. This will drastically increase the efficiency of virtual screening.

### Similarity conservation in latent space

When using a transformer model to auto-encode SMILES strings, the used loss function commonly only consists of a reconstruction term, e.g. in form of a cross entropy loss. While this may be sufficient to conserve similarities in latent space for small datasets, the model does not specifically learn relationships between molecules. The triplet loss function introduced in the previous section can be used to separate labelled samples in latent space. Since the herein presented work uses continuous data, a similarity threshold has to be defined with the intention of distinguishing between similar and dissimilar compounds. The determination of such a threshold is ambiguous and may differ between systems and their active molecules.

To better deal with the continuous nature of our data, we developed a novel loss function which we call the similarity loss (Eq. 4).

4 $$\begin{aligned} L(A, X) = \big | a\cdot \Vert (1-sim(A, X))\Vert - \Vert f(A) - f(X)\Vert \big | \end{aligned}$$

The similarity loss depends on an anchor (*A*) sample much like in the triplet loss function. However, it does not have to rely on the determination of positive and negative (i.e. similar and dissimilar) samples. Instead, it compares each anchor in a batch with all other samples (*X*) in the same batch. Since most similarity metrics $sim(\cdot ,\cdot )$ range from 0 to 1 (0 being completely different and 1 being identical), $1 - sim(\cdot ,\cdot )$ can be used to convert the similarity to a relative distance. The loss function is therefore trying to set the Euclidian distance in latent space equal to the relative distance in data space. In this study we used the Tanimoto coefficient calculated based on Morgan fingerprints as similarity metric. However, the described loss function is agnostic of the used similarity metric as long as its values are in the range [0, 1]. In order to spread the embedded samples in latent space, we included a scaling factor *a* to the term describing the relative distance in data space. The complete loss function consists of the sum of reconstruction loss (here we use a cross entropy loss) and our similarity loss:

5 $$\begin{aligned} \begin{aligned} L(A, X)\,=\, \big | a\cdot \Vert (1-sim(A, X))\Vert - \Vert f(A) - f(X)\Vert \big | - \sum _{I\in \{A,X\}}\sum _{i=1}^{n_I}\sum _{c}t_{i,c}\cdot log({\hat{p}}_{i,c}) \end{aligned} \end{aligned}$$

where $t_{i,c}$ is the label of a token *i*, ${\hat{p}}_{i,c}$ is the predicted probability for class *c* for token *i*, and $n_I$ is the number of tokens for compound *I*. More information about the training of the model such as the selection of anchors during the batch generation can be found in the Additional file 1: Section 2.1.

In the following subsections, we compare the performance of the presented loss functions in order to determine their suitability to conserve similarities in latent space.

## Results and discussion

### Initial tests using a small dataset

For a comparison of the three loss functions, three models were trained on a small dataset containing 10,000 compounds (see Additional file 1). The three models were trained using the reconstruction loss of SMILES strings (vanilla transformer), reconstruction plus triplet loss function, and reconstruction plus our newly developed similarity loss function. To compare the performance of the three models, we predicted the distances between a set of 100 randomly chosen reference compounds from the validation set and all other compounds in the dataset and compared them to the respective ground truth similarities. Based on these calculations, we computed the area under the receiver operating characteristics curve (AUROC) using different similarity thresholds to distinguish similar from dissimilar compounds. To avoid bias from the high number of dissimilar compounds leading to increased AUROC values, we only included compounds with a mimimum similarity of 0.40 to the individual reference compounds in this analysis.

**Table 1:** AUROC values for the different models trained on a small dataset of 10,000 compounds

Similarity threshold	Vanilla transformer	Triplet loss	Similarity loss
0.45	0.68 ± 0.17	0.73 ± 0.17	0.82 ± 0.18
0.50	0.69 ± 0.18	0.75 ± 0.16	0.86 ± 0.17
0.55	0.75 ± 0.18	0.80 ± 0.15	0.92 ± 0.08
0.60	0.76 ± 0.18	0.81 ± 0.15	0.91 ± 0.11
0.65	0.80 ± 0.17	0.85 ± 0.13	0.94 ± 0.09
0.70	0.84 ± 0.18	0.89 ± 0.12	0.96 ± 0.07
0.75	0.87 ± 0.16	0.91 ± 0.12	0.97 ± 0.07
0.80	0.90 ± 0.14	0.94 ± 0.09	0.98 ± 0.07
0.85	0.92 ± 0.14	0.96 ± 0.08	0.98 ± 0.07
0.90	0.94 ± 0.14	0.98 ± 0.05	0.98 ± 0.08
0.95	0.97 ± 0.09	0.99 ± 0.04	1.00 ± 0.01

While the vanilla transformer model was trained using only a reconstruction loss function, the other two models were trained with an additional loss term to specifically enforce the conservation of ground truth similarities in the latent space

As shown in Table 1, although there were overlapping error bands, the model trained with our similarity loss function in addition to the reconstruction loss clearly outperformed the other two models. The AUROC values were above 0.90 for all tested similarity thresholds except the lowest two. For all three methods, we observed an increase in AUROC values with increasing similarity threshold. This is likely due to a negative correlation between the true positive rate and the total number of positives in a dataset.

The vanilla model often failed to distinguish between similar and dissimilar compounds based on the Euclidian distances in latent space. The predicted distances are all very similar which likely caused a blurring in latent space, rendering it difficult to accurately distinguish between similar and dissimilar samples. While the model trained with an additional triplet loss was often able to map similar compounds closer to the reference than dissimilar compounds, it also generated a very dense latent space in which small errors can lead to incorrect predictions. By including our custom similarity loss, the model not only learned to correctly distinguish between similar and dissimilar molecules most of the times, it also spread out the generated latent space much more, making a separation between molecules much clearer.

**Fig. 3:**
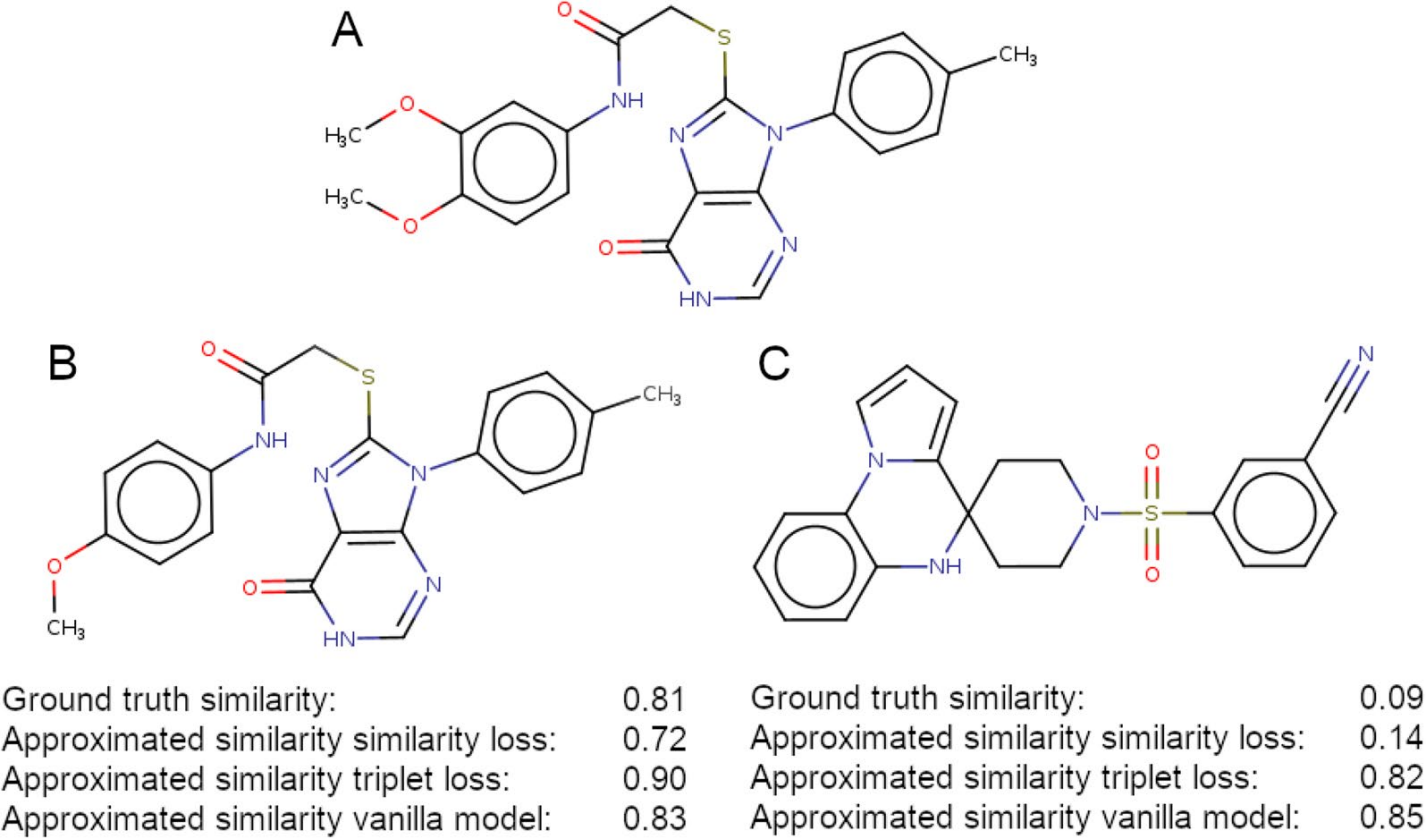
Similarity conservation in latent space. **A** 2D structure of a randomly chosen reference compound. **B** 2D structure of a molecule similar to the reference. Similarity was defined as having a Tanimoto coefficient above 0.8. The distances to the reference in latent space are shown for the individual models. **C** 2D structure of a dissimilar molecule. Dissimilarity was defined as having a Tanimoto coefficient below 0.3. Latent space distances to the reference are shown for the individual models

Figure 3 highlights the differences between the three models on a randomly selected example. Compound B is highly similar to compound A, whereas compound C does not share a high similarity with A. Scaling the latent space distance $d_{ij}$ between two molecules *i* and *j* to the range [0, 1] and translating them into similarities $s_{ij}^{LS}$, allows for a comparison of ground truth and predicted similarities in latent space:

6 $$\begin{aligned} \begin{aligned} s_{ij}^{LS} \approx 1-\frac{d_{ij}}{d_{max}}, \end{aligned} \end{aligned}$$

where $d_{max}$ is maximum distance between any two molecules in latent space.

By applying this formula to the compounds in Fig. 3, we obtain approximated similarities between A and B of 0.724, 0.899, and 0.825, and between A and C of 0.139, 0.821, and 0.852 using the similarity loss model, the triplet loss model, and the vanilla model, respectively. This shows that the similarity loss model is clearly better at discriminating between similar and dissimilar molecules.

While the vanilla transformer model has no additional information about the similarity between molecules, the triplet loss function learns to group similar molecules together based on a similarity threshold. In contrast, the similarity loss function directly maps similarities to Euclidian distances and thereby, a superiority in this specific task was expected.

Based on these results, we expected the model with the additional similarity loss function to perform best, followed by the model with the triplet loss. Since the vanilla model did not have the ability of explicitly learning to couple similarities with latent space distances, we expected it to perform worst in the similarity-based virtual screening tasks.

### Scale-up using the ZINC database

**Fig. 4:**
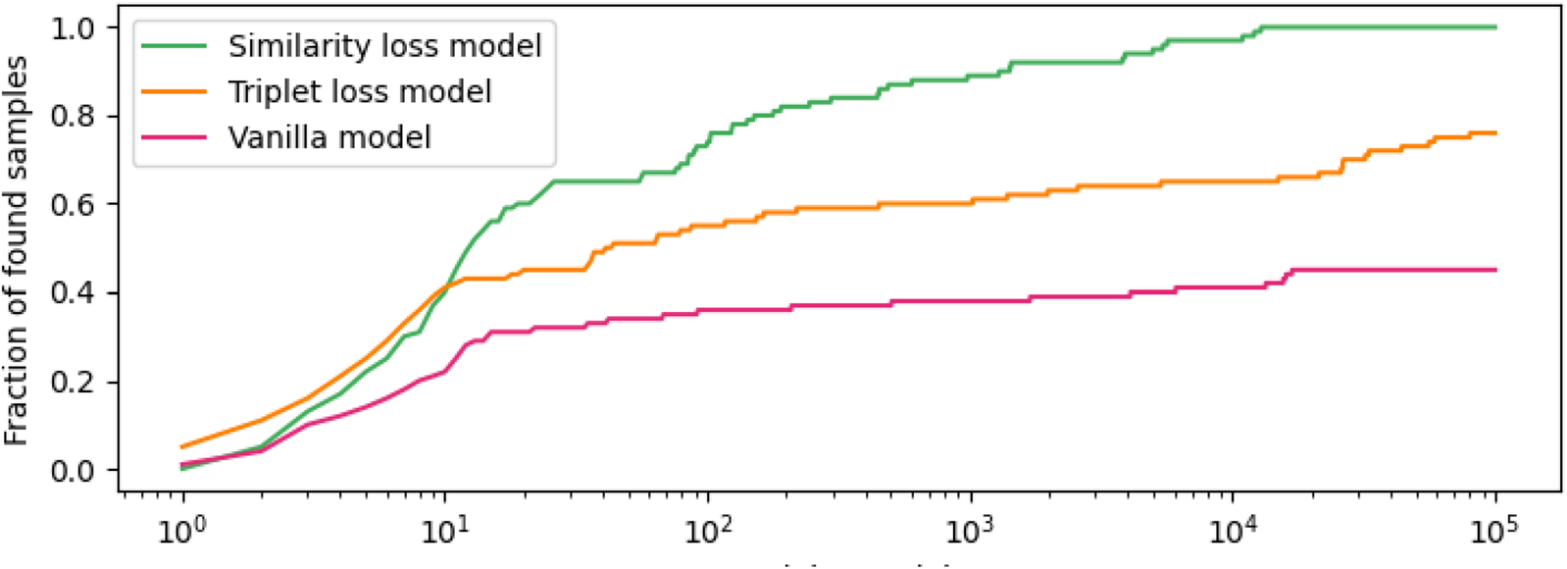
Comparison of reproduction abilities of the models with and without similarity loss function. The lines represent the normalized amount of the 10 most similar compounds within the top *N* closest samples in latent space for 10 reference compounds

Training of the models was subsequently upscaled using a large dataset of around 500,000 molecules (see Additional file 1: Dataset generation). To test the optimized model, we chose a diverse set of 10 reference compounds and screened the whole downloadable ZINC database (around 1.5 billion SMILES) against each reference compound [35]. The 10 reference compounds were randomly selected from the complete ZINC database while ensuring some degree of structural diversity and making sure that the compounds were neither part of the training nor the validation set. An overview of all 10 reference compounds can be found in Additional file 1: Fig. S4. The goal of these models was not to achieve a perfect correlation with calculated 2D similarities but to reduce the search space to a manageable size for subsequent exhaustive similarity search. We therefore checked for each reference compound how many of the 10 most similar database entries (determined using an exhaustive search) can be found within the *N* closest samples according to each model (Fig. 4).

The model trained with the similarity loss function proved to be effective in reproducing the top 10 most similar compounds within the 15,000 closest samples in latent space for all investigated reference compounds. This corresponds to a reduction of the search space by 5 orders of magnitude. In comparison, the vanilla model (i.e. without similarity loss function) only managed to identify 45% of all similar compounds within the top 100,000 predictions. With an identification rate of 75%, the model trained with the triplet loss was better than the vanilla model while still being worse than the model with similarity loss.

**Fig. 5:**
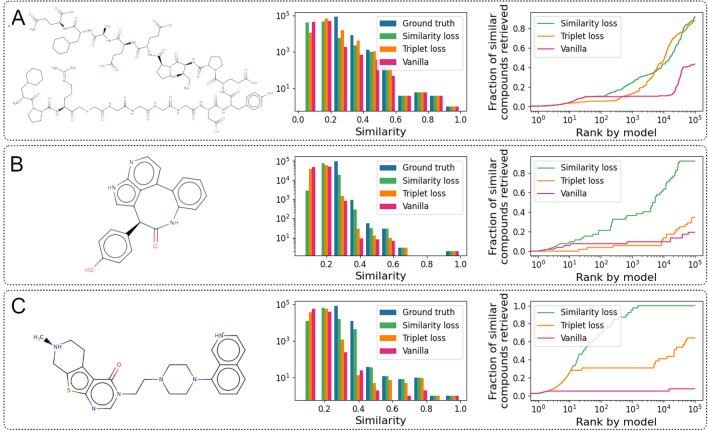
Similarity reproduction abilities. Left: 2D structure of the respective reference compound. Middle: Histogram of similarities (calculated using the exact method) of the 100,000 closest molecules to the reference in latent space (”ranking” task). Right: Reproduction of fairly similar compounds to the reference where a threshold of 0.5 was chosen to distinguish between similar and dissimilar compounds (”hit identification” task). **A** analysis of the performance using a very large reference compound. **B** performance with a smaller, cyclized reference compound. **C** performance using a more linear compound with heterocycles

To give further insights into the performance differences between the individual models, we selected three structurally different compounds from the 10 reference molecules. The first reference (**reference1**) is a large peptide with a molecular weight of more than 2000 g/mol (PubChem CID 44335764). The second (**reference2**) is a highly cyclized compound (PubChem CID 44605611) and the third (**reference3**) is a potent 5HT1B receptor antagonist (PubChem CID 44405730).

The first ”ranking” analysis (Fig. 5, middle column) shows the models’ potential to correctly identify and rank the 100,000 most similar compounds from the ZINC database. The right column in Fig. 5 analyses the models’ performance in identifying similar compounds to the reference (at a similarity threshold of 0.5). This analysis we name ”hit identification” in the subsequent paragraphs. In general, the vanilla transformer was capable to identify similar compounds to large reference molecules such as **reference1**, but had significant difficulties for small substances, e.g. **reference3**. The same was true for the triplet loss model although the reproduction performance for the small substances was better compared to the vanilla model (Fig. 5).

In detail, the analysis showed that all three models performed very well for **reference1** (Fig. 5A), with the triplet loss model being slightly better at reproducing the similarity distribution of the exact metric than the other two models. In the ”hit identification” task, with approximately the first 100 predictions, all models performed similarly. For the compounds ranked lower in predicted similarity to the reference, the similarity and triplet loss models started to clearly outperform the vanilla model. Within 100,000 top-ranked compounds, the similarity and triplet loss models were able to reproduce around 90% of the similar compounds whereas the vanilla model only managed to find around 40%.

For **reference2** (Fig. 5B) and **reference3** (Fig. 5C), the similarity loss model clearly outperformed the other two models in both ”ranking” and ”hit identification” tasks. For **reference2**, the similarity loss model, triplet loss model, and vanilla model were able to identify 90%, 33%, and 18% of the similar compounds, respectively. Large difference was also seen for **reference3**, where the similarity loss could identify all similar compounds within the top 2000 predictions while the vanilla model could only find around 7% of the similar compounds within the first 100,000 predictions. The triplet loss model was able to find 63% of the most similar compounds, thus performing much better than the vanilla model but still much worse than the model trained with the similarity loss. The comparatively good performance of the vanilla and triplet loss model for **reference1** is likely due to the relatively low number of very large molecules in the data set, placing those molecules in a well-separated location in latent space. The model trained on the similarity loss however performed well in all three cases, proving the advantage of the additional loss term.

#### Exclusion of scaling factor in loss function

To study the importance of the scaling factor in the similarity loss function (Eq. 4), we trained an additional model with a scaling factor of 1, thus disabling its effect. Using the same analyses as previously discussed revealed a drop in accuracy compared to using larger scaling factors, although it still performs better than the vanilla model (Additional file 1: Fig. S5). These findings have likely to do with the fact that a well structured latent space that is not too densely packed may be important for a good reproduction performance.

Finding a good value for the scaling factor is not trivial and this hyperparameter has to be tuned during training. In our tests, we found a value of 20 to work well for the initial analyses with a smaller dataset. However, when moving to a larger set, we found that decreasing the scaling factor to 10 further improves the performance of the model.

## Conclusion

In this work, we developed models for similarity-based high-content screening with the aim to translate pairwise similarities in data space to Euclidian distances in latent space. This will facilitate efficient similarity searches independent of similarity metrics. We could show that the use of a loss function specifically designed to conserve molecular similarities in latent space greatly improved the accuracy of the model. By training a transformer autoencoder using a novel similarity loss function, it was possible to obtain a model that could be successfully used for similarity search against a database of more than 1 billion compounds. We demonstrated that our model was able to generalize from a comparatively small dataset, making it possible to learn highly complex similarity metrics that could otherwise not be applied to large datasets. While the presented model did not obtain a perfect correlation to the underlying ground truth similarity metric, it can be used to substantially reduce the available search space by five orders of magnitude. Such a drastic reduction of search space allows for subsequent use of exhaustive classical screening methods.

Here, we provide a proof of concept showing the possibility of generating a model for similarity search that is unaware of the underlying similarity metric, thereby uncoupling its efficiency from the chosen method. For future adaptation of the method to 3D similarities, we will explore whether SMILES representations are sufficient as input or representations such as 3D graphs are necessary to allow the model to effectively learn 3D information. The proposed loss function for latent space shaping, however, will be not affected by this potential architecture change, as it is agnostic of the specific similarity metric.

## Supplementary Information

**Additional file 1.** Results and discussion section. **Figure S1.** Reproduction of molecular weights.** Figure S2.** Reproduction of molecular weights. Materials and methods section. **Figure S3.** Example of SMILES tokenization. **Figure S4.** All reference compounds used for the assessment of the reproduction ability. **Figure S5.** Performance of the model trained with the similarity loss scaling factor set to 1 forthe ”hit identification” task.

## Data Availability

The code used to train the model and screen the database can be found on GitHub (https://github.com/mmodbasel/HighContentScreening).
