# Supplementary material for: Efficient virtual high-content screening using a distance-aware transformer model
Source: J Cheminform. 2023 Feb 8;15:18. doi: 10.1186/s13321-023-00686-z (PMC9906956; doi:10.1186/s13321-023-00686-z)
Supplement: Supplementary file 1 — Additional file 1. Results and discussion section. Figure S1. Reproduction of molecular weights. Figure S2. Reproduction of molecular weights. Materials and methods section. Figure S3. Example of SMILES tokenization. Figure S4. All reference compounds used for the assessment of the reproduction ability. Figure S5. Performance of the model trained with the similarity loss scaling factor set to 1 forthe ”hit identification” task. [file 13321_2023_686_MOESM1_ESM.pdf]

Additional File 1: Efficient Virtual High-Content Screening Using a Distance-Aware Transformer Model

Manuel S. Sellner, Amr H. Mahmoud, and Markus A. Lill*

Computational Pharmacy, Department of Pharmaceutical Sciences, University of Basel, Klingelbergstrasse 50, 4056 Basel, Switzerland

E-mail: markus.lill@unibas.ch

1. Additional Results and Discussion

To further investigate the reproduction abilities of the model with and without similarity loss, we analyzed the distribution of molecular weights of the 100,000 molecules predicted to be closest to the reference (Figure S1).

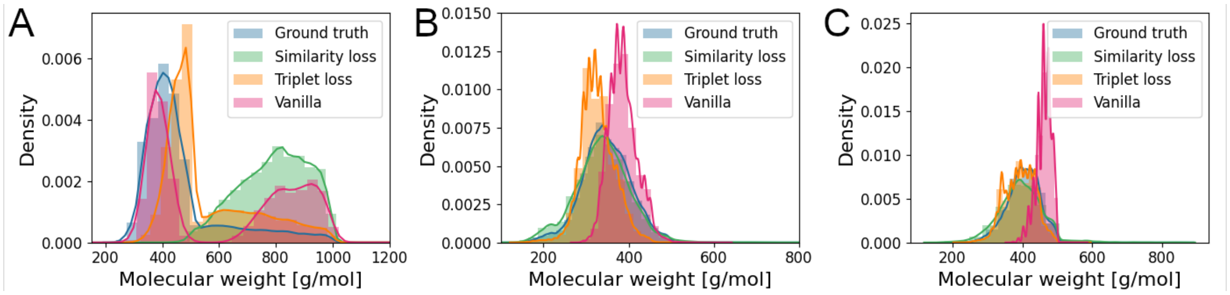

Figure S1: Reproduction of molecular weights. The histograms show the distribution of molecular weights of the 100,000 most similar compounds to **reference1** (A), **reference2** (B), and **reference3** (C) calculated using either the exact similarity metric, the model with similarity loss, or the vanilla transformer model.

The data show that all three models are well able to reproduce the molecular weight distribution of the 100,000 most similar compounds to **reference1** while the triplet loss model outperforms the other two models. This effect is the most pronounced at the lower end of the scale where the vanilla and triplet loss models are able to reproduce more of the low molecular weight compounds than the similarity loss model. More detailed analysis of this phenomenon revealed that these low molecular weight compounds are all highly dissimilar to the reference compound. When only including compounds with a similarity to **reference1** of 0.3 or more, these compounds disappeared and the similarity loss model showed a better overlap with the ground truth. Still, the triplet loss model showed a slightly better reproduction of the molecular weights than the other two models (Figure S2). The sampling of very dissimilar molecules may be due to the fact that the vanilla and triplet loss models generated a much denser latent space, leading to a generally lower distance between the very high molecular weight compounds and the molecules with lower molecular weight. While this benefits the two models for **reference1**, it decreases their performance for **reference2** and **reference3** (Figure S1 B & C). In these examples, the model with similarity loss is generally better able to reproduce the distribution of molecular weights from the underlying (exact) similarity metric. Here, the vanilla transformer model is likely suffering because there are a lot of molecules in the screened data set that have a similar molecular weight to the two reference compounds. This causes the model to over-sample these compounds in the densely packed latent space. In these cases, the sparser latent space generated by the similarity loss may prevent such an over-sampling.

For **reference 2**, the triplet loss model performed similarly to the vanilla model (Figure S1B). However, for **reference 3** it appears that the triplet loss model has about the same performance as the similarity loss model (Figure S1C). Here, it must be noted that while the 100,000 sampled compounds have a similar distribution of the molecular weight, only 96 had a similarity of at least 0.3 to the reference compound (compared to 8,236 for the similarity loss model, Figure S2C). Thus, while in some cases the triplet loss model is able to

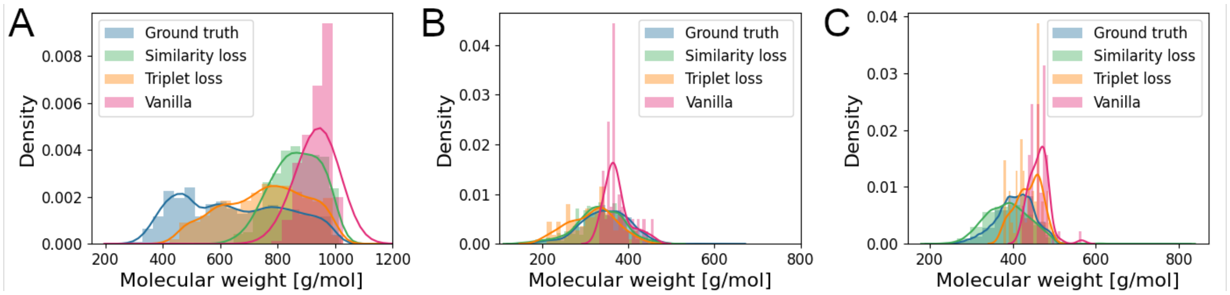

Figure S2: Reproduction of molecular weights. The histograms show the distribution of molecular weights of the 100,000 most similar compounds to **reference1** (A), **reference2** (B), and **reference3** (C) calculated using either the exact similarity metric, the model with similarity loss, or the vanilla transformer model. Only compounds with a similarity of at least 0.3 are considered.

nicely reproduce compounds with a similar molecular weight compared to the ground truth, it still lacks the ability to find compounds with high structural similarity to the reference.

2. Additional Materials and Methods

The following section will describe the detailed neural network architecture, its hyperparameters, and the datasets used to train and test the model.

2.1 SMILES Transformer

Our model uses a transformer architecture as described in the publication by.[S1] It was implemented in PyTorch using their integration of the Transformer module. The vocabulary was generated using tokenized SMILES strings that were used as input and encoded into 256 dimensional latent space. Our model consisted of 4 encoder and decoder layers with attention layers containing 4 heads. All models were trained using an Adam optimizer with a learning rate of 10^{-4} and 128 samples per batch. Since it was not possible to further increase the batch size due to memory limitations, we accumulated the gradients over 4 batches.

In order to determine the ground truth similarities, we calculated the Tanimoto coefficients based on 1024 bit Morgan fingerprints implemented in RDKit with a radius of 2.

To conserve similarities in latent space, it is imperative that during training, each batch contains at least one similar compound to each sample (and for the triplet loss also at least one dissimilar compound). For the model trained on the similarity loss, we first randomly assigned compounds to a batch which act as anchor. To guarantee that similar compounds exist for each of those reference compounds, the algorithm randomly selected 3 of the 100 most similar compounds to the reference which were added to the batch. For the model with the triplet loss, we randomly selected 64 anchors per batch and for each chose a random compound with a Tanimoto similarity to the anchor of at least 0.6. It was assumed, that due to the intrinsic diversity of the dataset, for each anchor in a batch, there will always be a negative sample present. We defined negative samples as any compound with a similarity of less than 0.4 to the anchor.

The scaling factor a required by the similarity loss function was set to 20.0 in the initial tests on a small dataset and was later decreased to 10.0 for the scaled up training. The margin m for the triplet loss function was set to 1.0 for the comparison of the loss functions as well as for the scaled up model. These values were determined based on the retrospective analysis of the performance of each trained model.

Training a model with the similarity loss and the hyperparameters described above for 1000 epochs took roughly 9 days on a single GTX 1080 Ti.

2.1.1 Datasets

During an initial test phase, we used a randomly selected subset of 10,000 SMILES extracted from the natural compounds dataset obtained from the ZINC database. The dataset was randomly split into a training (80%) and validation (20%) set. The validation set was used to compare the performances of three different loss functions. In the upscaling experiments, we randomly selected 0.03% of the compounds in each tranche downloaded from the ZINC database, leading to a dataset consisting of approx. 500,000 compounds. Following the method of the initial test, the dataset was randomly split into a training and validiation set

using a 80/20 split. For testing the optimized model, the whole ZINC database was used which consisted of around 1,458,000,000 compounds at the time of testing.

For reproducibility, all used SMILES strings were converted to their canonical form using openbabel prior to training and testing.

2.2 Similarity Search

Once obtained, the distance aware SMILES embeddings were used to efficiently calculate distances (i.e. similarities) in embedding space. Facebook's faiss was utilized for this task using a FlatL2 index to calculate Euclidian distances in latent space. Faiss allows the construction and search of several types of indexes with various degrees of approximation.

The search was performed on pre-calculated latent space embeddings of the whole ZINC database. Searching 94 reference compounds against the complete database took roughly 2.75 hours on a machine with 64GB RAM that was equipped with an HDD. Around 65% of the computation time was needed to read the pre-computed embeddings from disk. By using either a server with solid state drives or more memory, the computational cost could therefore be significantly decreased. Searching the same database using RDKit's BulkTanimotoSimilarity function (with pre-computed fingerprints) on the same machine required around 3.40 hours for a single reference compound.

3. Additional Figures

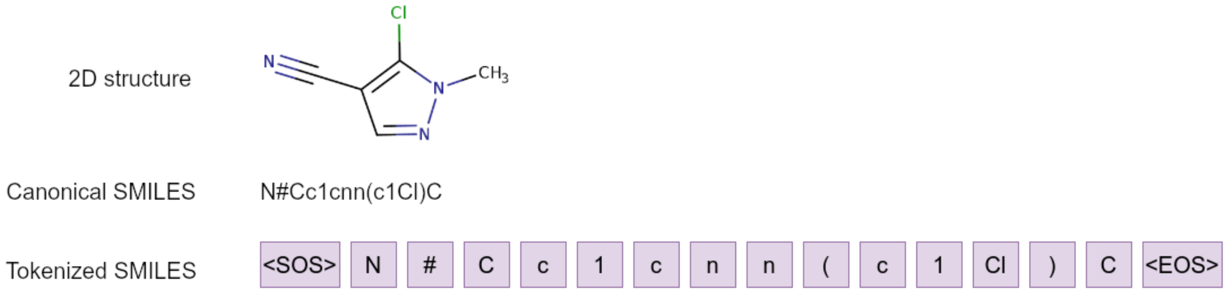

Figure S3: Example of SMILES tokenization. The 2D structure of a molecule, its SMILES representation, and the tokenized SMILES are shown. ”<SOS>” and ”<EOS>” represent labels specifying the start and the end of the sequence, respectively.

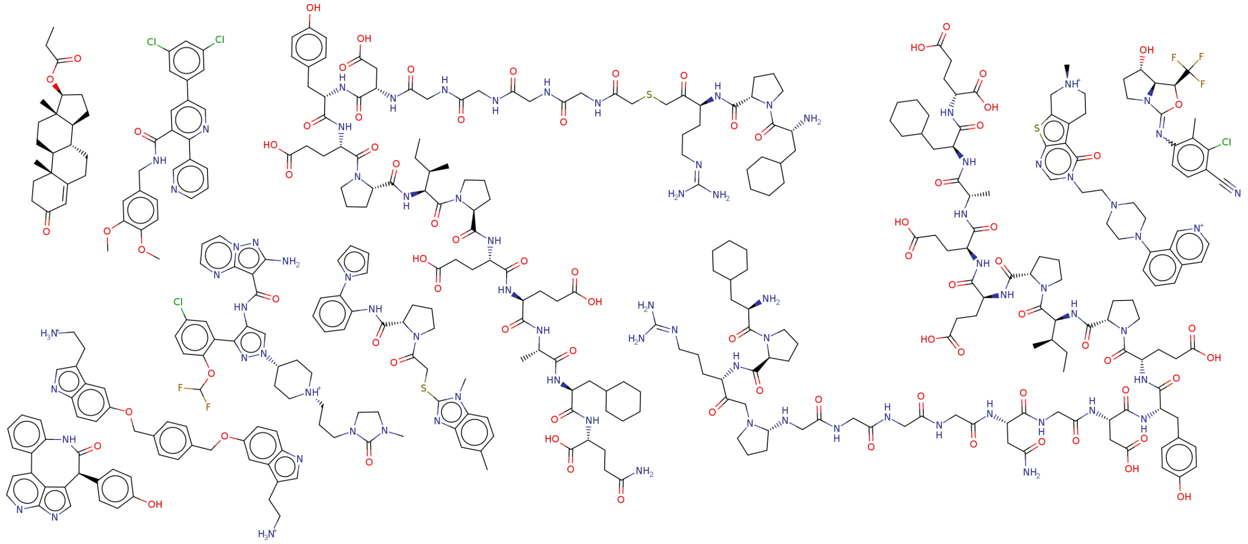

Figure S4: All reference compounds used for the assessment of the reproduction ability.

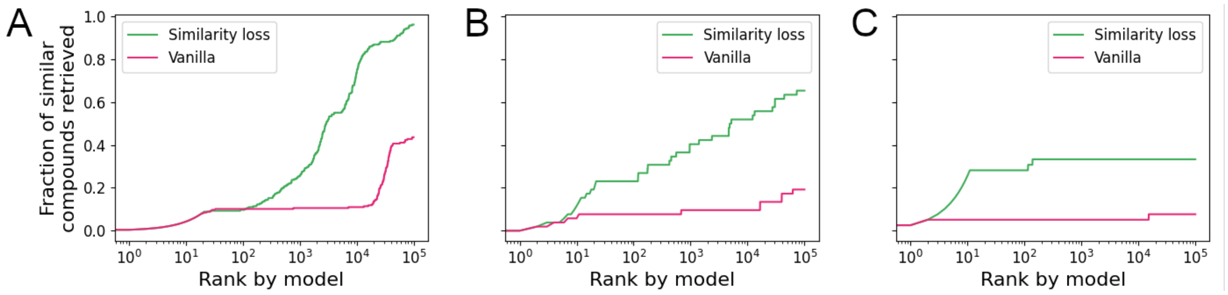

Figure S5: Performance of the model trained with the similarity loss scaling factor set to 1 for the ”hit identification” task. The data for **reference1** (A), **reference2** (B), and **reference3** (C) are shown.

References

(S1) Vaswani, A.; Shazeer, N.; Parmar, N.; Uszkoreit, J.; Jones, L.; Gomez, A. N.; Kaiser, Ł.; Polosukhin, I. Attention is all you need. Advances in Neural Information Processing Systems. 2017; pp 5999–6009.
